# Sex Differences in Desiccation Tolerance Varies by Colony in the Mesic Liverwort *Plagiochila porelloides*

**DOI:** 10.3390/plants11040478

**Published:** 2022-02-10

**Authors:** Juliana da C. Silva-e-Costa, Andrea P. Luizi-Ponzo, David Nicholas McLetchie

**Affiliations:** 1 Department of Botany, Federal University of Juiz de Fora, Juiz de Fora 36036-900, MG, Brazil; luizi.ponzo@ufjf.edu.br; 2 Department of Biology, University of Kentucky, Lexington, KY 40506-0225, USA; mclet@uky.edu

**Keywords:** desiccation tolerance, ecophysiology, environmental association, net carbon assimilation, maximum quantum yield, sex difference

## Abstract

Water scarcity, a common stress factor, negatively impacts plant performance. Strategies to cope with it, such as desiccation tolerance, are becoming increasingly important to investigate. However, phenomena, such as intraspecific variation in stress responses have not received much attention. Knowledge of this variability and the environmental drivers can be leveraged to further investigate the mechanisms of desiccation tolerance. Here we tested for variation in desiccation tolerance in *Plagiochila porelloides* among colonies and sexes within the same riparian zone. Field-collected dehardened plants were subjected to a desiccation event, under controlled conditions and then rehydrated. Plant water status, photosynthetic rates, net carbon gain, and efficiency of photosystem II (PSII) were assayed to evaluate tissue desiccation, basic metabolic processes and plant recovery. To establish a linkage between plant response and environmental factors, field light conditions were measured. We detected intraspecific variation, where a more exposed colony (high percentage of open sky, large temporal range of light quantity, and high red/far-red ratio) showed sex differences in desiccation tolerance and recovery. Overall, PSII recovery occurred by 72 h after rehydration, with a positive carbon gain occurring by day 30. This within species variation suggests plastic or genetic effects, and likely association with light conditions.

## 1. Introduction

Water scarcity is amongst the most common abiotic stress factors that influence crop productivity [1,2], and it is an important ecological aspect and an evolutionary driver in terrestrial environments [3]. In order to cope with water stress, plants evolved several strategies, including desiccation tolerance, a trait found in many spores, seeds and pollen, and also in vegetative tissue [3,4,5,6]. In general, desiccation tolerance can be defined as the ability to dry out to equilibrium with the surrounding air to an absolute water content (WC) of <−100 MPa [7,8], which could be as low as 0.1 g H_2_O g^−1^ dry weight or 10% [9], and then return to normal metabolic activity after rewetting [3,10,11]. 

Desiccation tolerance is an important mechanism to ecological and evolutionary responses, particularly in photosynthetic tissue of resurrection plants (including bryophytes) [9]. In spite of an extensive body of research on desiccation tolerance, including several recent reviews [6,9,12,13,14], there are substantial gaps in the literature on intraspecific desiccation tolerance. Most mechanistic driven studies involved few model species or do not evaluate local adaptation or plasticity of species; such evaluations have the potential of being leveraged to promote a better understanding of plant biology in the face of climate change [15].

Sex differences in water stress, as an intraspecific variability, are found in seed plants but the sex that is more tolerant varies greatly across species [16,17,18]. Male and female plants in species with unisexual individuals may have different physiologies, due to sex function [19], and these might include (either directly or indirectly) different desiccation tolerance responses. In the few bryophyte species studied where a sex difference occurred or was indicated, females were found to be more water stress tolerant than males or have responses that were interpreted as more tolerant [20,21,22,23,24]. 

Species that are desiccation-tolerant are often associated with regions (deserts and inselbergs) that experience very dry periods [25]. Some functional traits in bryophytes, such as life forms, are driven by environmental water and light gradients [26]. Within species, an association between desiccation tolerance and environmental factors is rarely made. In fact, few studies have tested for intraspecific differences in desiccation [15] (and references therein). For example, the percentage of canopy openness (exposure) was related with increased male water stress tolerance [24] and UV light was positively associated with tolerance [27]. Identifying and quantifying environmental associations to desiccation tolerance will provide insight into selection drivers or their correlates. 

Here we use the species *Plagiochila porelloides* (Torr. ex Nees) Lindenb., a leafy liverwort, to test for variation in desiccation tolerance among three colonies and between the sexes along stream edges where microhabitats variation might occur. *Plagiochila* is a genus of worldwide distribution, with ~700 species recognized [28,29]. At least two species of *Plagiochila* have been reported as desiccation-tolerant; these studies focused on documenting the physiological response to a desiccation event [30,31,32,33,34] and not intraspecific variation in desiccation tolerance. Using *P. porelloides*, this study: (1) describes light conditions (including exposure) of the studied colonies, (2) tests for differences in desiccation tolerance among colonies, and (3) tests for differences in desiccation tolerance between male and female plants. We predict that colony light conditions (including exposure) can influence the desiccation tolerance response because higher light conditions can result in lower moisture conditions and thus selection for higher water stress tolerance relative to lower light conditions; a previous study found a positive relationship between exposure and water stress tolerance (dehydration tolerance) [24]. We expect that more exposed colonies will contain more desiccation-tolerant plants. We also predict that females will have a higher desiccation tolerance level than males, as previously detected in other bryophytes. We found that desiccation tolerance differed by colony and sex and the pattern of desiccation tolerance was associated with light parameters. 

## 2. Material & Methods

### 2.1. Study Organism, Sampling Conditions and Field Characteristics 

*Plagiochila porelloides* is a leafy liverwort with unisexual individuals, found in Asia [35], Europe [36,37] and North America [38,39]. The common growth substratum for *P*. *porelloides* is moist boulders, generally in mesic (shady and moist) environments [35,37]. *Plagiochila porelloides* has remarkable flexibility to edaphic conditions and occurs under a wide range of moisture and light conditions [40] but it is often identified as a shade-loving species [41,42].

Species identification was verified using the Plagiochilaceae key of North America [40]. Specimens were vouchered at the Missouri Botanical Garden Herbarium—MO (St Louis, MO, USA—Silva-e-Costa, J.C. 135 & D.N. McLetchie) and duplicates were sent to Morehead State University Herbarium—MDKY (Morehead, KY, USA). Plants for this study were collected on large boulders along streams of Robinson Forest Research Station (Department of Forest, Agriculture College, The University of Kentucky), Clayhole, Breathitt County, Kentucky, USA, in two sites: Clemons Fork and Big MillSeat. 

Sex expressing plants were used for this study; the sexes were identified in the field because male and female plants present distinct sex structures. These structures were observed with a 10× hand lens and the plants were collected. Plants were taken into the laboratory to confirm field sex and species identification, using a dissecting microscope (10× to 50×). We collected twelve male and twelve female plants from each of three different boulders (henceforth referred to as colonies), physically separated by streamflow, and at least ten meters from each other. These colonies were chosen based on the abundance of sex expressing plants. Colonies were geopositioned (GPSMAP^®^ 62sc, Garmin International, Inc., Olathe, KS, USA) and marked: Colony 1 (C1): 37°28′44″ N, 83°8′56″ W–Big MillSeat stream, Colony 2 (C2): 37°28′18″ N, 83°8′42″ W–Clemons Fork stream, and Colony 3 (C3): 37°28′18″ N, 83°8′43″ W–Clemons Fork stream. Plants were kept for at least one week after collection in common conditions (see below) to allow for dehardening [9].

In order to describe the general air moisture conditions of *P*. *porelloides* colonies along the stream’s edges, we collected field data of temperature and relative humidity (RH), using a humidity sensor HOBO ^TM^ placed within two cm of the surface of plants and attached to a data logger (Onset Computer Corporation, Bourne, MA, USA). To estimate the more extreme water demanding conditions as will occur during the daytime, data were recorded at 5 min intervals between 10:00 to and 15:00 h (GTM-5) at least twice a month for each month from August to November 2017 on various colonies along the streams. We report the range of means of temperature, RH and vapor pressure deficit. 

To describe the general light measures of each colony we estimated the percentage of canopy openness (variable that indicates exposure) [24] and light quantity (photosynthetic flux density (PPFD, mol m^−2^ day^−1^)) using hemispherical canopy photographs taken on 2–9 August 2017 with a Coolpix 4500, (Nikon, Tokyo, Japan) with a 180° fisheye lens attached. The digital photographs were analyzed with WinSCANOPY (Regent Instruments, Ville de Québec, QC, Canada) to assess the light environment. WinSCANOPY uses the known path of the sun (daily and seasonally), the longitudinal and latitudinal of where the picture was taken, and the open sky of the hemispherical photograph to calculate PPFD. We estimated PPFD every 5 days for a period from 1 May to 28 September, which represents the period where the canopy was leafed out (the USA National Phenology Network, www.usanpn.org—accessed on 21 December 2017). To measure light quality (red to far red ratio, which decreases as light passes through vegetation) reaching the studied colonies on a cloud-free day, between 12:00 and 14:30 h, on 7 September 2017 (trees with leaves), we used a JAZ spectrophotometer calibrated with a LS-1-CAL light source (Ocean Optics, Dunedin, FL, USA). For each colony, we report the values for each light variable.

### 2.2. Desiccation Tolerance Assay and Recovery of Photosystem II

All experiments were conducted in a recovery environmental chamber (Percival^®^ Model T-35LL) with a 12 h light/12 h dark cycle period, light condition ranging between 24 to 44 µmol m^−2^ s^−1^ (GE fluorescent cool white lamps, 20 Watt), and a constant temperature of 14 °C. 

The desiccation assays involve a drying event for hydrated plants and a rewetting event. To determine a minimum endurable relative humidity (RH) for desiccation tolerance and the rewetting protocol, we completed a pilot experiment using salt solutions to achieve 75% (Sodium Chloride), 55% (Magnesium Nitrate) and 33% (Magnesium Chloride) RH [43]. Sufficient salt was added to each salt solution so that salt crystals were visible in the solution during the experiment. We also tested rewetting protocols for recovery using (1) liquid water or a (2) pre-hydration period [9] for 6 h in high RH prior to the addition of liquid water. Pre-hydration conditions were created by removing the saturated salt solution and adding liquid water inside the chamber (a quantity sufficient to cover the bottom of the chamber), while the plants were kept on the dried filter paper in Petri dishes; this process precedes the addition of liquid water on plant tissue. *Plagiochila porelloides* were able to survive at the lowest RH (Magnesium Chloride) tested and recovered better in pre-hydration conditions compared to direct rewetting to achieve rehydration. Thus, we used Magnesium Chloride salt solution (which measured a value of 40%RH with a humidity sensor HOBO^TM^) and pre-hydration to test for variation in desiccation tolerance. 

**Figure 1:**
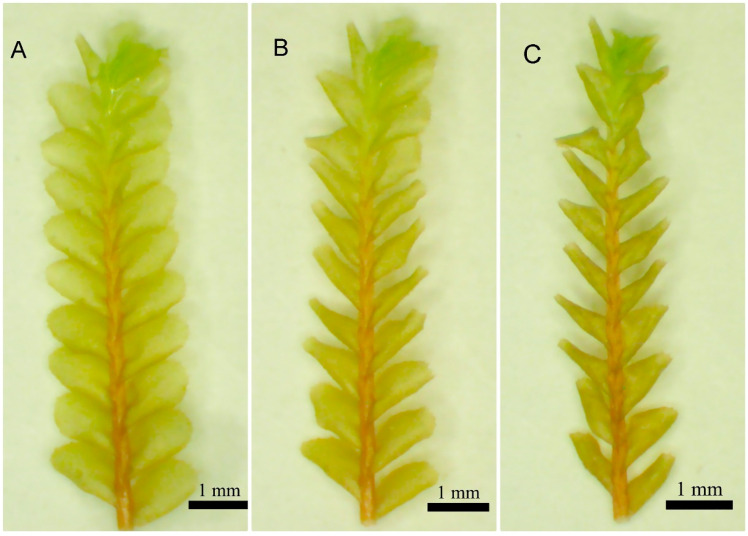
*Plagiochila porelloides* morphology visually changes with hydration status–dorsal view. (**A**) full hydrated condition, with no liquid water added; (**B**) partly desiccated condition; (**C**) completely dry condition, exhibiting highly contorted leaves.

Prior to the desiccation assays, plants were placed in full hydrated conditions for 24 h (Figure 1A). Plants were then blotted to remove external water, using filter paper (No. 1 Whatman^®^ filter paper) and immediately placed in 35 × 10 mm Falcon^TM^ Petri dishes with 125 µL of distilled water added to the filter paper. This condition provides a slow drying process that avoids severe physiological damage [9,44,45]. 

Following the experimental design described in [22], Petri dishes containing male and female plants were placed inside four desiccation chambers, each containing three males and three females from each colony, for a total of 18 plants per desiccation chamber. Assignment to a desiccation chamber was random. RH inside the chamber during desiccation was verified using the humidity sensor HOBO^TM^ attached to a data logger (Onset Computer Corporation, Bourne, MA, USA). Plants were kept in desiccation conditions for 22 h.

To test for the status of the plants, we assayed for maximal quantum yield ($F_{v}/F_{m}$) of photosystem II (PSII) as a proxy for recovery. $F_{v}/F_{m}$ is routinely used to assay and indicate recovery in desiccation-tolerant studies [9,44] and references therein). To measure $F_{v}/F_{m}$, we used an OS5p+-FL modulated chlorophyll fluorometer (Opti-Sciences, Tyngsboro, MA, USA) with measurement parameters set to a saturation intensity of 100 (4000 µmol m^−2^ s^−1^) and a pulse duration of 0.8 s. Prior to data collection, plants were dark-adapted for 20 min. To determine initial status, $F_{v}/F_{m}$ were measured on all plants before the desiccation event. After the desiccation period of 22 h, plants were placed in pre-hydration conditions, for a period of six hours. Immediately after this high humidity exposure, liquid water was added to the plant and, again, the efficiency of PSII was measured at various intervals: 0 h, 4 h, 12 h, 24 h, 48 h, 72 h, 144 h, and 216 h. The following equation was used to calculate percent recovery for each plant for each recovery interval.

$$\mathrm{Percent}\;\mathrm{recovery}=(\frac{F_{v}/F_{m}\;\mathrm{at}\;\mathrm{time}\;t\;}{{\mathrm{Initial}\;F}_{v}/F_{m}})\times 100$$

### 2.3. Evaluation of Water Content by Dry Weight 

To evaluate shoot water content (WC) at 40% RH and to establish the rate of drying, ten vegetative plants of *P. porelloides* previously maintained in the environmental chamber were subjected to a desiccation protocol as described above. For this assay, plants were placed on a single 40% RH desiccation chamber. To assay WC and plant water lost, we weighed each plant to the nearest microgram using a Chan 29 electrobalance, after 14 h, 18 h, and 22 h of desiccation. To obtain dry weight (DW), plants were then oven-dried at 70 °C for 48 h. The first time of weighting (14 h) was determined by observing the desiccated leaves morphology (curled or contorted leaves, Figure 1B,C) [9]. If the mass did not change in value between the weights, we considered that the plant tissue was equilibrated to RH in the desiccation chamber. The WC was calculated on a dry weight basis [9], as shown below:

$$\mathrm{WC}\left(\%\right)=\frac{\left(\mathrm{Shoot}\;\mathrm{mass}\;\mathrm{at}\;\mathrm{RH}-\mathrm{DW}\right)}{\mathrm{DW}}\times 100$$

### 2.4. Gas Exchange Responses to Desiccation 

To confirm that desiccation reduced key physiological activities and that recovery resulted in a return of these activities we measured gas exchange (in light and in dark) in haphazardly collected plants from the field. These plants underwent three different treatments. Because *P. porelloides* plants are small, we measured three shoots (a set) at a time to increase the gas exchange signal. The treatment groups were G1—seven sets of plants that were not desiccated, G2—three sets of desiccated plants, measured three hours after rewetting, and G3—three sets of plants measured three days (72 h) after rewetting. Finally, groups G2 and G3 were re-assayed one month after desiccation.

Gas exchange was measured with an open-flow system (LI-6800, Li-Cor, Lincoln, NE, USA) equipped with a multiphase flash fluorometer as the light source. In the sample chamber, air temperature was set to 24 °C and relative humidity at 75% (vapor pressure deficit; VPD = 0.749 kPa). The airflow rate was set at 125 µmol s^−1^, and the reference CO_2_ was set at 450 µmol mol^−1^. In a pilot trial, we determined that maximum assimilation (A_max_) for *P. porelloides* occurred by a light level of 100 µmol m^−2^ s^−1^. Thus, physiological activity in the light was tested at 100 µmol m^−2^ s^−1^. The infrared gas analyzers (sample and reference) were matched prior to assays. 

The sample area of the three plants per set was estimated by measuring the length and width of each shoot, assuming the shoot is rectangular, and the leaves cover this area (Figure 1A). Plants were placed on acetate and held in place with a thin clear fishing line (0.2 mm thick). To standardize water status across assays, plants were blotted dry with tissue paper to remove free water and 5 µL of water were added to ensure that plants had enough moisture to prevent drying out before the completion of the assay. CO_2_ assimilation rates (µmol CO_2_ m^−2^ s^−1^) were taken after equilibrium (approximately 6 min after the beginning of the assay) and at 100 µmol m^−2^ s^−1^ (net photosynthesis) and 0 µmol m^−2^ s^−1^ (respiration in the dark). Generally, three readings were taken within one minute to obtain a mean estimate for that sample set. These means were used for statistical analysis.

### 2.5. Statistical Analyses

We used a combination of Microsoft Excel (2016) and JMP^®^ 12 (SAS Institute, Cary, NC, USA) to analyze the data. We considered *p* < 0.05 as significant.

#### 2.5.1. Desiccation Tolerance Assay 

Using percent recovery, which was arcsine transformed to improve normality [46], we evaluate colony and sex effects over time, performing a repeated measures analysis using multivariate analysis of variance (MANOVA) with a full factorial design. To perform the test, we used colony and sex as independent variables in the full factorial model effect (Colony, Sex and Colony by Sex), and evaluated: (1) Time, (2) Time × Colony, (3) Time × Sex, and (4) Time × Colony × Sex as possible interactions. Time was evaluated to test if plants increased in recovery, significant main effects (colony and sex) would indicate that colonies and the sexes differed in recovery. Interaction of these main effects over time would indicate that differences in the main factor depended on time. 

#### 2.5.2. Evaluation of Water Content by Dry Weight

To assess water content by dried weight, descriptive statistical analyses were performed, and graphs were made to visualize the relationship between water loss and time in the desiccation chamber.

#### 2.5.3. Gas Exchange in the Light in Response to Desiccation

To test for recovery differences in photosynthesis (gas exchange at 100 µmol m^−2^ s^−1^), we used an ANOVA and then used the contrast option of JMP^®^ to compare the mean photosynthesis of plants that were not desiccated with the mean photosynthesis of plants that were desiccated and in recovery after hydration for three hours, 72 h and one month. 

#### 2.5.4. Gas Exchange in the Dark in Response to Desiccation 

To test for recovery differences in respiration (gas exchange at 0 µmol m^−2^ s^−1^), we used an ANOVA and then used the contrast option of JMP^®^ to compare the mean respiration of plants that were not desiccated and the mean respiration of plants that were desiccated and in recovery after hydration for three hours, 72 h and one month. 

## 3. Results

### 3.1. Field Environmental Conditions 

**Figure 2:**
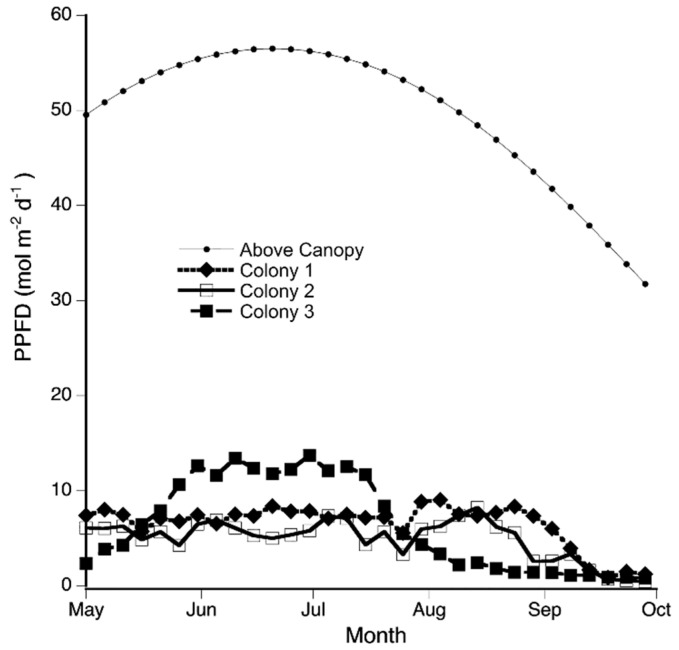
Colony 3 received more light than colony 1 and 2. From late May to the middle of July, *Plagiochila porelloides* colony C3 was estimated to receive more light than the two other *P. porelloides* colonies (PPFD below 9 mol m^−2^ d^−1^). Otherwise, colony C3 received similar or lower PPFD than the other two colonies. PPFD was estimated every 5 days from May to September using hemispherical canopy photographs and analyzed with WinSCANOPY. Above the canopy is the estimated PPFD above the canopy which was the same for all three colonies. C1, C2, and C3 are colonies 1, 2 and 3.

As an overall description of moisture conditions of the site during the day for the period of August to November, *P. porelloides* experienced mean field RH ranging from 59.61% to 94.2% (mild and high humidity levels), and a mean temperature ranging from 4.10 °C, in late autumn, to 22.54 °C, during the summer. These RHs and temperatures translated to vapor pressure deficit (VPD) that ranged from 0.0623 to 0.9880 kPa. Colonies C1 and C2 had similar canopy openness (6.54 and 6.49%, respectively) while colony C3 had almost twice the canopy openness (11.3%). PPFD above the canopy was the same for all three colonies and ranged from a high of 56 mol m^−2^ d^−1^ in June to a low of 32 mol m^−2^ d^−1^ in September. However, for two consecutive months, PPFD reaching colony C3 was between 10 and 14 mol m^−2^ d^−1^ (19.5% to 24.4% of full sunlight) while PPFD for the other two colonies was always below 9 mol m^−2^ d^−1^ (14.9% of full sunlight) (Figure 2). The high light level reaching colony C3 coincided with the increased light intensity above the canopy (Figure 2). Mean PPFD reaching the colonies for the overall period ranged from 4.95 ± 0.38 for C2 to 6.50 ± 0.40 for C1 (Table 1). On cloudless days, colonies C1 and C2 had similar red to far-red ratios (0.19 and 0.24) while colony C3 had almost twice the red to far-red ratio (0.46) (Table 1). 

### 3.2. Desiccation Tolerance Recovery

#### 3.2.1. Main Effects

**Figure 3:**
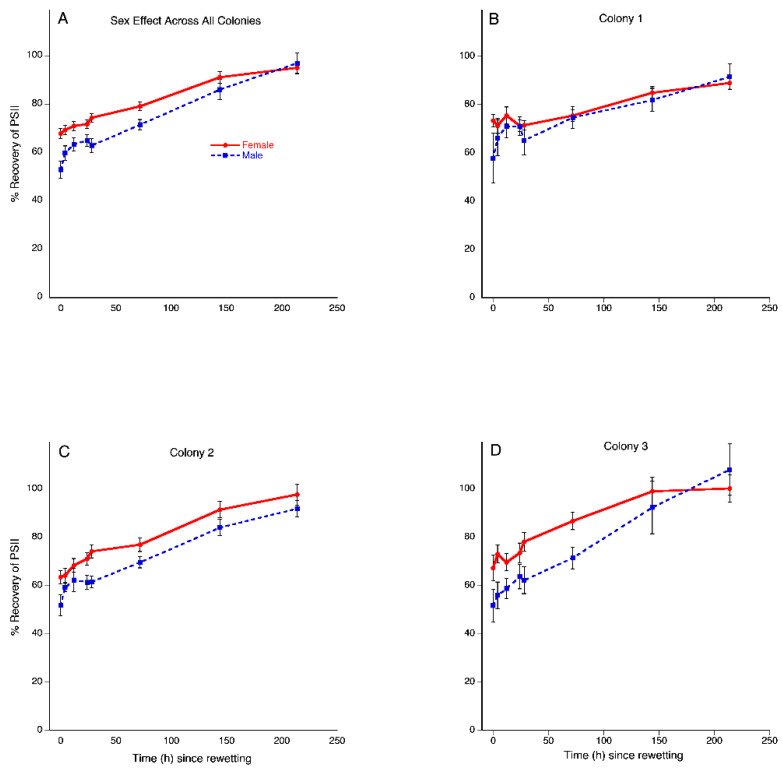
Desiccation tolerance in *Plagiochila porelloides* differs by sex and varies across colonies. Percent recovery of photosystem II assayed by chlorophyll fluorescence over 216 h after the drying event. (**A**) Overall sex difference in recovery for all three colonies combined; (**B**,**C**) Colony 1 and 2 respectively, showed no sex differences in recovery, although colony 2 was approaching a tendency of females having higher recovery (*p* = 0.1113). (**D**) Colony 3 showed higher recovery in females relative to males. Data are untransformed percent recovery. The bars indicate standard errors.

There was an overall time effect, indicating significant increases in plant recovery (F = 35.5669 _7/20_, *p* < 0.0001). Overall, colonies did not statistically differ in recovery (F = 0.2353 _2/26_, *p* = 0.1983). Generally, females had statistically higher recovery than males, as demonstrated by an overall sex effect (F = 4.7408 _1/26_, *p* = 0.0387) (Figure 3A).

#### 3.2.2. Time × Colony Interaction Effect

Colony recovery over time statistically differed as indicated by a significant colony by time interaction (F = 2.5559 _14/40_, *p* < 0.01). 

#### 3.2.3. Time × Sex Interaction Effect

The difference in male and female recovery did not statistically differ over time as indicated by a non-significant sex by time interaction (F = 1.1083 _7/20_, *p* = 0.3958). 

#### 3.2.4. Time × Colony × Sex Interaction Effect 

There was a significant time by colony by sex interaction (F = 2.0737 _14/40_, *p* = 0.0360), indicating that the difference between the sexes varied by colony. To further explore this interaction, each colony was analyzed separately to test for sex differences. Recovery of males and females did not statistically differ in colonies C1 (F = 1.4956 _7/5_, *p* = 0.4882; Figure 3B) and C2 (F = 29.1519 _7/2_, *p* = 0.1113; Figure 3C), but females had a statistically higher percentage of recovery than males in C3 (F = 39.8416 _1/7_, *p* < 0.001; Figure 3D).

### 3.3. Water Content by Dry Weight

**Figure 4:**
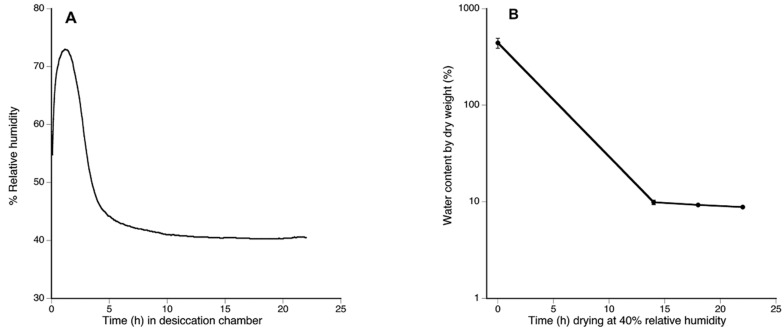
By 14 h in the desiccation chamber *Plagiochila porelloides* plants reached a desiccated state. (**A**) Relative humidity (RH) inside desiccation chamber during the assay showing RH stabilized in 40% after 10 h (600 min); (**B**) Water plant status showing water content loss during the desiccation assay at 40% RH. The bars indicate standard errors.

After six hours, RH in desiccation chambers approached 40%, and remained stable until the end of the experiment (Figure 4A). At this RH and temperature 14 °C (environmental chamber temperature), the VPD value was 0.9309 kPa. At the start of the assay, turgid tissue had a WC of 442.28 ± 136.49% (Figure 4B); tissue equilibrated to the desiccation conditions had a WC below 10% (9.87 ± 1.24% by 14 h; 9.27 ± 0.50% by 18 h, and 8.81 ± 0.33% by 22 h (Figure 4B). 

### 3.4. Gas Exchange Responses to Desiccation

**Figure 5:**
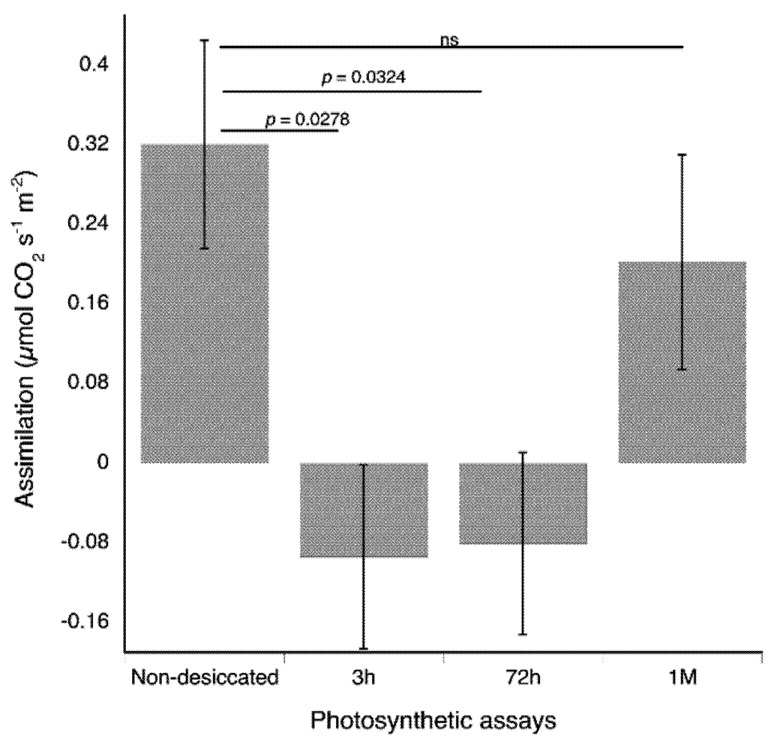
Non-desiccated *Plagiochila porelloides* plants have higher net carbon gain than plants rehydrated for three hours and 72 h but not for one month (M). Net carbon assimilation was evaluated through photosynthetic assays in three treatment groups (G1—seven sets of non-desiccated plants, G2—three sets of desiccated plants, measured three hours after rehydration, and G3—three sets of desiccated plants measured three days (72 h) after rehydration. G2 and G3 were again measured one month after rehydration. The bars indicate standard errors.

The photosynthetic rates of non-desiccated plants were 0.3205 ± 0.1047 µmol CO_2_ s^−1^ m^−2^. Desiccated plants in the recovering process for 3 h and 72 h had a net carbon loss, with a photosynthetic rate of −0.0938 ± 0.0922 µmol CO_2_ s^−1^ m^−2^, and −0.0805 ± 0.0912 µmol CO_2_ s^−1^ m^−2^, respectively. These rates were significantly lower than the rate for non-desiccated plants (t = 2.4367, *p* = 0.0278 and t = 2.358, *p* = 0.0324 for comparisons between non-desiccated and recovering plants at 3 h and 72 h respectively). After a month of recovering in hydrated conditions, the photosynthetic rates of plants that were recovering was 0.2021 ± 0.1079 µmol CO_2_ s^−1^ m^−2^. This assimilation rate was not statistically different from the photosynthetic rate of non-desiccated plants (t = 0.8636, *p* = 0.4014) (Figure 5). The dark respiration rates of plants did not statistically differ among non-desiccated and desiccated plants (*p* values ranged from 0.3152 to 0.4634). 

## 4. Discussion

Desiccation tolerance of the mesic shade liverwort *Plagiochila porelloides* differed by colony and by sex. This pattern of desiccation tolerance might be associated with light parameters. While photosystem II recovered after a few days, this recovery did not immediately translate to a net gain in carbon. 

### 4.1. Colony Specific Responses and Environmental Associations 

The variation in desiccation tolerance was primarily driven by one colony where a sex specific response was detected. This phenotypic variation would be due to a genetic or a plastic response. Plants in this study were dehardened for at least seven days and it is expected that this process would have removed field effects [9]. Thus, this result indicates a genetic component to this phenotypic variation. Recently, Ref. [24] suggested that genetic variation among individuals within species can explain fluctuating sex differences in water stress tolerance. In addition, phenotypic variation due to plastic responses has been linked to culturing methods [47,48] and life stages [49]. 

The effects of the environment on the desiccation tolerance response in bryophytes is well known [44,50] but some influences of the environment are less appreciated. For example, those authors suggest that bryophyte responses to desiccation are due to previous drought intervals and different intensities of water deficit. However, few studies on desiccation tolerance incorporate multiple locations and attempt to provide linkage with environmental variables at those locations [15]. 

The bryophyte plant body is at the scale of centimeters, and thus, the microclimate at this scale will be critical for the ecophysiology of an individual [44]. This microclimate will easily vary across the landscape, modulating the phenotypic responses of desiccation tolerance in a species [44] and potentially result in different adaptive responses across the landscape [24]. In this study’s attempt to provide a linkage between desiccation tolerance and habitat, we found evidence that the C3 colony was more exposed (more canopy openness) and had a high red/far red ratio–which is consistent with being a less shaded site-than the other two colonies. Additionally, C3 received high light intensity for two consecutive months. We speculate that the light parameters measured in this study or environmental correlates of these measures can affect the desiccation tolerance response. The presence of a causal relationship needs further study.

Recently, a study found that ultraviolet light resulted in an increase in desiccation tolerance in *Syntrichia caninervis* Mitt. [27]. While UV light was not measured in this current study, the pattern of light measures of C3 vs. C1 and C2 translates to more direct light and thus more UV light for C3. However, the direction of the relationship might not be transferable across species. For example, a sex difference in water stress tolerance that varied by exposure (canopy openness) was reported for vegetative plants in the liverwort *Marchantia inflexa* Nees & Mont. [24]. Interestingly, the sex difference was found in a less exposed site and not the more exposed sites as found in this current study. Regardless of the relationship, within species variation can be leveraged to better understand the nuances of desiccation tolerance response that will provide valuable source material to study the molecular causes of the variation in this response, including the roles of local adaptation and plasticity in this trait [15]. 

### 4.2. Sex Differences on Water Stress Tolerance

Here we found that PSII efficiency $(F_{v}/F_{m}$) of female plants of *P. porelloides* recovered more from a desiccation event than males, but eventually, both recovered to pre desiccation levels. Reproductive traits are greatly influenced by drought [13]. Previous authors demonstrated and discussed sex differences under water stress [51,52,53,54]; male and female plants, especially while sex-expressing, may exhibit distinct growth rates [51], reproductive investment [52,53] and water holding capacity [54].

Female advantages for desiccation tolerance have been previously suggested and documented in bryophytes [20,21,22,23] (see [53] for a lack of sex difference). Further, female reproductive investment prior to and after fertilization likely requires higher efficient water use and a higher stress tolerance than males [51,55].

Sex differences in water stress responses are reported for seed plants and can vary depending on species, environmental conditions [18], and which sex is more tolerant [56,57,58,59,60]. Thus, sex responses to water limitations and their consequences can differ across diverse lineages [15].

### 4.3. Physiological Responses to Recovery on Rehydration Processes

Bryophytes often experience many drying/rewetting cycles during their life span [10,61]. In these cycles, there is a net carbon loss after rehydration [61]. Although the photosystems can recover within minutes of rewetting [62,63], respiration recommences even quicker [64], causing an initial carbon loss for recovering plants. 

Our results showed that assessment of recovery based on quantum yield versus carbon gain was different: photosystem II recovery occurred in three days, while carbon gain recovery was complete after a longer period of rehydration. Thus, initial photosystem II recovery did not reflect carbon gain. Similar results were found in the mosses *Anomodon viticulosus* (Hedw.) Hook. & Taylor [65] and in *Syntrichia caninervis* Mitt. [66], where net photosynthesis was negative immediately after rewetting, reflecting increased respiration rates and a carbon deficit state. 

In conclusion, we found that *P. porelloides* has intraspecific variation in desiccation tolerance. This variation was due mostly to one colony, where the sexes differed. We suggest that the variation had a genetic component and might be due to variation in light. In order to provide more comprehensive insights of sex differences in *P. porelloides,* future studies on desiccation tolerance variation should include vegetative plants.

## Figures and Tables

**Table 1:** Light related measures (canopy openness, PPFD and red to far-red ratio) and sex differences in desiccation tolerance among colonies.

Colony	Canopy Openness (%)	PPFD ± se (mol m^−2^ d^−1^)	Red/Far Red Ratio	Sex Difference in Desiccation Tolerance
C1	6.54	6.50 ± 0.40	0.19	ns
C2	6.49	4.95 ± 0.38	0.24	ns
C3	11.30	6.35± 0.87	0.46	F > M (*p* < 0.001)

## Data Availability

The data presented in this study are openly available in [Figshare] at [https://doi.org/10.6084/m9.figshare.14079791], reference number [14079791].

## References

[1] Osakabe Y., Kawaoka A., Nishikubo N., Osakabe K. (2011). Responses to environmental stresses in woody plants: Key to survive and longevity. J. Plant Res..

[2] Smirnoff N. (2014). Plant Stress Physiology. Encyclopedia of Life Science.

[3] Alpert P. (2005). The limits and frontiers of desiccation-tolerant life. Integr. Comp. Biol..

[4] Alpert P., Oliver M.J., Black M., Pritchard H.W. (2002). Drying without dying. Desiccation and Survival in Plants: Drying without Dying.

[5] Farrant J.M., Lehner A., Cooper K., Wiswedel S. (2009). Desiccation tolerance in the vegetative tissues of the fern *Mohria caffrorum* is seasonally regulated. Plant J..

[6] Zhang Q., Bartels D. (2018). Molecular responses to dehydration and desiccation in desiccation-tolerant angiosperm plants. J. Exp. Bot..

[7] Bewley J.D. (1979). Physiological aspects of desiccation tolerance. Ann. Rev. Plant Physiol..

[8] Proctor M.C.F., Pence V., Black M., Pritchard H.W. (2002). Vegetative Tissues: Bryophytes, vascular resurrection plants and vegetative propagules. Desiccation and Survival in Plants: Drying without Dying.

[9] Stark L.R. (2017). Ecology of desiccation tolerance in bryophytes: A conceptual framework and methodology. Bryologist.

[10] Oliver M.J., Shaw J.A., Goffinet B. (2009). Biochemical and molecular mechanisms of desiccation tolerance in bryophytes. Bryophyte Biology.

[11] Vanderpoorten A., Goffinet B. (2009). Introduction to Bryophyte Biology.

[12] Lüttge U., Beck E., Bartels D. (2011). Plant Desiccation Tolerance.

[13] Vitt D.H., Crandall-Stotler B.J., Wood A.J., Rajakaruna N., Boyd N.R., Harris T. (2014). Survival in a dry world through avoidance and tolerance. Plant Ecology and Evolution in Harsh Environments.

[14] Oliver M.J., Farrant J.M., Hilhorst H.W.M., Mundree S., Williams B., Bewley J.D. (2020). Desiccation Tolerance: Avoiding Cellular Damage During Drying and Rehydration. Annu. Rev. Plant Biol..

[15] Marks R.A., Farrant J.M., Mcletchie D.N., VanBuren R. (2021). Unexplored dimensions of variability in vegetative desiccation tolerance. Am. J. Bot..

[16] Freeman D.C., McArthur E.D. (1982). A comparison of twig water stress between males and females of six species of desert shrubs. For. Sci..

[17] Sinclair J., Emlen J., Freeman D.C. (2012). Biased Sex Ratios in Plants: Theory and Trends. Bot. Rev..

[18] Juvany M., Munné-Bosch S. (2015). Sex-related differences in stress tolerance in dioecious plants: A critical appraisal in a physiological context. J. Exp. Bot..

[19] Delph L.F., Geber M.A., Dawson T.E., Delph L.F. (1999). Sexual Dimorphism in Life History. Gender and Sexual Dimorphism in Flowering Plants.

[20] Newton M.E. (1972). Sex-ratio differences in *Mnium hornum* Hedw. and *M. undulatum* Sw. in relation to spore germination and vegetative regeneration. Ann. Bot..

[21] Stieha C.R., Middleton A.R., Stieha J.K., Trott S.H., Mcletchie D.N. (2014). The dispersal process of asexual propagules and the contribution to population persistence in *Marchantia inflexa* (Marchantiaceae). Am. J. Bot..

[22] Marks R.A., Burton J.F., McLetchie D.N. (2016). Sex differences and plasticity in dehydration tolerance: Insight from a tropical liverwort. Ann. Bot..

[23] Slate M., Rosenstiel T.N., Eppley S.M. (2017). Sex-specif.f.fic morphological and physiological differences in the moss *Ceratodon purpureus* (Dicranales). Ann. Bot..

[24] Marks R.A., Pike B., McLetchie D.N. (2019). Water stress tolerance tracks environmental exposure and exhibits a fluctuating sexual dimorphism in a tropical liverwort. Oecologia.

[25] Alpert P. (2000). The discovery, scope and puzzle of desiccation tolerance in plants. Plant Ecol..

[26] Giordani P., Incerti G., Rizzi G., Rellini I., Nimis P.L., Modenesi P. (2013). Functional traits of cryptogams in Mediterranean ecosystems are driven bywater, light and substrate interactions. J. Veg. Sci..

[27] Ekwealor J.T.B., Clark T.A., Dautermann O., Russell A., Ebrahimi S., Stark L.R., Niyogi K.K., Mishler B.D. (2021). Natural UV exposure alters photosynthetic biology and improves recovery from desiccation in a desert moss. J. Exp. Bot..

[28] Söderström L., Hagborg A., Von Konrat M. (2015). Notes on early land plants today. 69. Circumscription of Plagiochilaceae (Marchantiophyta) with a preliminary infrageneric subdivision of Plagiochila. Phytotaxa.

[29] Söderström L., Hagborg A., von Konrat M., Bartholomew-Began S., Bell D., Briscoe L., Brown E., Cargill D.C., Costa D.P., Crandall-Stotler B.J. (2016). World checklist of hornworts and liverworts. PhytoKeys.

[30] Clausen E. (1952). Hepatics and humidity, a study on the occurrence of hepatics in a Danish tract and the influence of relative humidity on their distribution. Dansk Bot. Ark..

[31] Dilks T.J.K., Proctor M.C.F. (1974). The pattern of recovery of bryophytes after desiccation. J. Bryol..

[32] Dilks T.J.K., Proctor M.C.F. (1976). Seasonal variation in desiccation tolerance in some British bryophytes. J. Bryol..

[33] Gupta R.K. (1976). The Physiology of the Desiccation Resistance in Bryophytes: Nature of Organic Compounds Leaked from Desiccated Liverwort, *Plagiochila asplenioides*. Biochem. Physiol. Pflanzen.

[34] Wood A.J. (2007). The nature and distribution of vegetative desiccation-tolerance in hornworts, liverworts and mosses. Bryologist.

[35] Söderström L., Rycroft D.S., Cole W.J., Wei S. (1999). *Plagiochila porelloides* (Plagiochilaceae, Hepaticae) form Changbai Mountain, new to China, with chemical characterization and chromosomes measurements. Bryobrothera.

[36] Cronberg N. (2000). Absence of genetic variation in populations of the liverwort *Plagiochila porelloides* from northern Greece and southern Scandinavia. Lindbergia.

[37] Sim-Sim M., Carvalho S., Fontinha S., Lobo C., Garcia C. (2003). *Plagiochila porelloides* (Torr. ex Nees) Lindenb. in mainland Portugal and Madera archipelago. New records and the threatened status. Port. Acta Biol..

[38] Stotler R.E., Cradall-Stotler B.J. (1997). A Checklist of the Liverworts and Hornworts of North America. Bryologist.

[39] Stotler R.E., Cradall-Stotler B.J. (2017). A Synopsis of the liverwort flora of north America north and Mexico. Ann. Mo. Bot. Gard..

[40] Schuster R.M. (1980). The Hepaticae and Anthocerotae of North America East of the Hundredth Meridian. Vol. IV.

[41] Mägdefrau K., Smith A.J.E. (1982). Life-forms of Bryophytes. Bryophyte Ecology.

[42] Gradstein S.R., Churchill S.P., Salazar-Allen N. (2001). Guide to the Bryophytes of Tropical America.

[43] Greenspan L. (1977). Humidity Fixed Points of Binary Saturated Aqueous Solutions. J. Res. Natl. Bur. Stand..

[44] Proctor M.C.F., Oliver M.J., Wood A.J., Stark L.R., Cleavitt N.L., Mishler B.D. (2007). Desiccation-tolerance in bryophytes: A review. Bryologist.

[45] Schonbeck M.W., Bewley J.D. (1981). Responses of the moss *Tortula ruralis* to desiccation treatments. II. Variations in desiccation tolerance. Botany.

[46] Sokal R.R., Rohlf J. (1995). Biometry: The Principles and Practice of Statistics in Biological Research.

[47] Stark L.R., Greenwood J.L., Brinda J.C., Oliver M.M.J. (2014). Physiological history may mask the inherent inducible desiccation tolerance strategy of the desert moss *Crossidium crassinerve*. Plant Biol..

[48] Brinda J.C., Stark L.R., Clarck T.A., Greenwood J.L. (2016). Embryos of a moss can be hardened to desiccation tolerance: Effects of rate of drying on the timeline of recovery and dehardening in *Aloina ambigua* (Pottiaceae). Ann. Bot..

[49] Stark L.R., Oliver M.J., Mishler B.D., McLetchie D.N. (2007). Generational differences in response to desiccation stress in the desert moss *Tortula inermis*. Ann. Bot..

[50] Proctor M.C.F., Shaw J.A., Goffinet B. (2009). Physiological Ecology. Bryophyte Biology.

[51] Haig D. (2016). Living together and living apart: The sexual lives of bryophytes. Philos. Trans. R. Soc. B.

[52] Stark L.R., Mishler B.D., McLetchie D.N. (2000). The cost of realized sexual reproduction: Assessing patterns of reproductive allocation and sporophyte abortion in a desert moss. Am. J. Bot..

[53] Stark L.R., Nichols L., McLetchie D.N., Bonine M.L. (2005). Do the sexes of the desert moss *Syntrichia caninervis* differ in desiccation tolerance: A leaf regeneration assay. Int. J. Plant Sci..

[54] Moore J., Kollar L., McLetchie D.N. (2016). Does selection for gamete dispersal and capture lead to a sex difference in clump water-holding capacity?. Am. J. Bot..

[55] Stark L.R., Greenwood J.L., Brinda J.C., Oliver M.J. (2013). The desert moss *Pterygoneurum lamellatum* (Pottiaceae) exhibits an inducible ecological strategy of desiccation tolerance: Effects of rate of drying on shoot damage and regeneration. Am. J. Bot..

[56] Leigh A., Nicotra A.B. (2003). Sexual dimorphism in reproductive allocation and water use efficiency in *Maireana pyramidata* (Chenopodiaceae), a dioecious, semi-arid shrub. Aust. J. Bot..

[57] Li C., Ren J., Luo J., Lu R. (2004). Sex-specific physiological and growth responses to water stress in *Hippophae rhamnoides* L. population. Acta Physiol. Plant.

[58] Álvarez-Cansino L., Zunzunegui M., Díaz-Barradas M.C., Esquivias M.P. (2010). Physiological performance and xylem water isotopic composition underlie gender-specific responses in the dioecious shrub *Corema album*. Physiol. Plant.

[59] Chen L., Zhang S., Zhao H., Korpelainen H., Li C. (2010). Sex-related adaptive responses to interaction of drought and salinity in *Populus yunnanensis*. Plant Cell Environ..

[60] Zhang S., Chen F., Peng S., Ma W., Korpelainen H., Li C. (2010). Comparative physiological, ultrastructural and proteomic analyses reveal sexual differences in the responses of *Populus cathayana* under drought stress. Proteomics.

[61] Mishler B.D., Oliver M.J., Knight C.D., Perroud P.F., Cove D.J. (2009). Putting *Physcomitrella Patens* on the tree of life: The evolution and ecology of mosses. The Moss Physcomitrella Patens.

[62] Proctor M.C.F., Smirnoff N. (2000). Rapid recovery of photosystems on rewetting desiccation-tolerant mosses: Chlorophyll fluorescence and inhibitor experiments. J. Exp. Bot..

[63] Proctor M.C.F. (2001). Patterns of desiccation tolerance and recovery in bryophytes. J. Plant Growth Regul..

[64] Oliver M.J. (1991). Influence of protoplasmic water loss on the control of protein synthesis in the desiccation-tolerant moss *Tortula ruralis*: Ramifications for a repair-based mechanism of desiccation tolerance. Plant Phys..

[65] Hinshiri H.N., Proctor M.C.F. (1971). The effect of desiccation on subsequent assimilation and respiration of the bryophytes *Anomodon viticulosus* and *Porella platyphylla*. New Phytol..

[66] Coe K.K., Belnap J., Sparks J.P. (2012). Precipitation-driven carbon balance controls survivorship of desert biocrust mosses. Ecology.

